# Phosphorus in the Treatment of Phthisis

**Published:** 1859-03

**Authors:** I. Gee

**Affiliations:** Salisbury Centre, N. Y.


[From the Boston Medical and Surgical Journal.]

*Phosphorus in the Treatment of Phthisis.*

Messers. Editors,—As Dr. Churchill's treatment of consumption has awakenen some interest, permit me to offer a few hints on the subject.

The distinguished doctor names a decrease of phosphorus in the system as the proximate cause of tubercle, and a supply of this deficiency as a cure. Upon this hint he has acted, and his experiment has given prominence to a medicine which will doubtless hereafter rank high as an adjuvant in exhaustive diseases and nervous debility (thus indirectly affecting phthisis,) but not it is feared as a cure or preventive of tubercle "specifically."

Will the "cured" cases of Dr. Churchill be exempt from phthisis in the future? This will be a test. That the treatment in these cases owes its success to phosphorus, will not be disputed. But the hypophosphites are not alone in their effect upon tuberculosis. Many sources of relief are chronicled. In fact, "flattering phases" are common records in the history of this disease, resulting often in a cure, but not a permanent immunity from the tubercular deposit. Admitting the good qualities of phosphorus, will its increase in the system be a permanent augmentation, which it must be to ensure lasting benefit.

Dr. Churchill has introduced a good medicine; but I am not inclined to believe he has solved the enigma of the tubercular diathesis.

And here let me broach a thought which for years I have entertained. The strongest evidence and the best testimony point to *debility* as the solution of tubercle. Consumption is incompatible with vigor of system. From the infirm portion of the race (frail families), the army of comsumptives is recruited. When exhaustive diseases reach a certain point of debility, tubercle appears. By the term debility, I do not mean general weakness—as the most feeble are sometimes exempt from phthisis—but a disproportionate enervation of the parts affected to those free from disease, and that enervation confined to the absorbents. This, then, seems to me a solution to the pulmonary deposit —*a lack of absorbent power in the lungs*—low in comparison to that of the other organs of the body, giving rise to the expressions "weak lungs," "feeble respiration," &c.

It is known that tubercles are sometimes absorbed; and this occurs when the lungs are in a reparative condition. Had this favorable condition prevailed at the time of the deposition, it is easy to conceive the tubercles would have been arrested, or absorbed in the act of deposition. I consider tubercles refuse matter not wanted in the system, but deposited from an inability of the absorbents to carry it off. If this is so, the remedy is palpable—increase the pulmonary energy. Why is the jolting wagon and horseback exercise recommended to consumptives? Ostensibly to increase the general vigor and health of the patient; but, really, *passive* exercise acts upon the *viscera*, or internal organs, which are beyond the reach of voluntary agitation.—Hence the resorts to this species of exercise in phthisis. Dr. Parrish, of Philadelphia, is a conspicuous example of the success of this mode of treatment. The inward organs of the body are feeble compared to the arms and legs which are toughened and strengthened by exercise, and hence free, or nearly so, from tubercles. Submit the viscera to a proper action, and you will restore the lost proportion and regain the stength necessary to prevent a refuse deposit. This *disproportion is greater in those predisposed to consumption than in those free from the "taint"* in whom the absorbent energy seldom reaches the low point of enervation which induces the deposit. I. GEE.

*Salisbury Centre, N. Y., Dec.* 3 1858.

